# Angioinvasive rhinocerebral mucormycosis with complete unilateral thrombosis of internal carotid artery—case report and review of literature

**DOI:** 10.1259/bjrcr.20150448

**Published:** 2016-05-01

**Authors:** Aruna Patil, Himansu Shekhar Mohanty, Sharath Kumar, Shrivalli Nandikoor, Prabhu Meganathan

**Affiliations:** ^1^ Department of Radiology, Apollo Hospitals, Bangalore, India; ^2^ Department of Pathology, Apollo Hospitals, Bangalore, India

## Abstract

Angioinvasive rhinocerebral mucormycosis is an acute fulminant infection caused by fungi of the order Mucorales that targets uncontrolled diabetics and other immunosupressed individuals. Early imaging features of angioinvasiveness include the presence of thrombosed vessels, especially in the orbital regions and the “black turbinate” sign representing devitalized paranasal sinus mucosa. Intracranial extension carries a grave prognosis, with death reported in 90% of cases. This case report highlights the early and key imaging features of angioinvasive rhinocerebral mucormycosis and a rare complication of complete internal carotid artery thrombosis.

## Summary

Angioinvasive rhinocerebral mucormycosis is an acute fulminant infection caused by fungi of the order Mucorales that targets uncontrolled diabetics and other immunosupressed individuals. Early imaging features of angioinvasiveness include the presence of thrombosed vessels, especially in the orbital regions and the “black turbinate” sign representing devitalized mucosa. Complete thrombosis of internal carotid artery (ICA), a rare complication, can add to higher morbidity rates.

## Clinical presentation

A 51-year-old female presented with recent onset mild headache and epistaxis to the otorhinolaryngology outpatient department. She was a known diabetic and hypertensive for 8 years, on irregular treatment.

**Figure 1.**
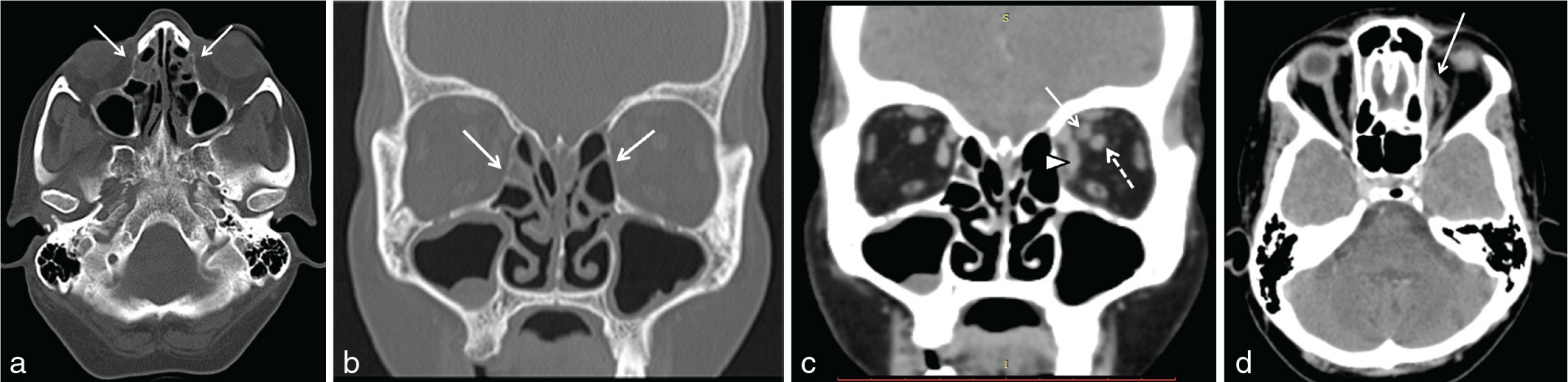
(a, b) Axial and coronal plain CT scans in bone window showing mucosal thickening of the bilateral ethmoidal and maxillary sinuses (arrows). (c, d) Contrast-enhanced CT scan in the coronal and axial sections showing linear non-enhancing soft tissue (arrows) located superomedial to the optic nerve (broken arrow). The thickening of the medial rectus muscle and the surrounding fat stranding (arrowhead) is significant.

Contrast-enhanced CT scan of the paranasal sinuses was perfmormed using 100 ml of Iohexol (Omnipaque 300 mgl ml^–1^; GE Healthcare, Shanghai, China) (Figure 1a,b), which showed moderate mucosal thickening of the paranasal sinuses, with no obvious hyperdense contents within the sinuses. The left orbit showed thickening of the medial rectus and superior oblique muscles with mild surrounding fat stranding. A linear non-enhancing structure with surrounding fat oedema was seen in the left orbit, related superomedially to the optic nerve (Figure 1c,d). It was continuous with the ophthalmic artery anteriorly and posteriorly, suggestive of ophthalmic artery thrombosis. No base of skull bony erosions or intracranial soft tissue extension were seen.

**Figure 2.**
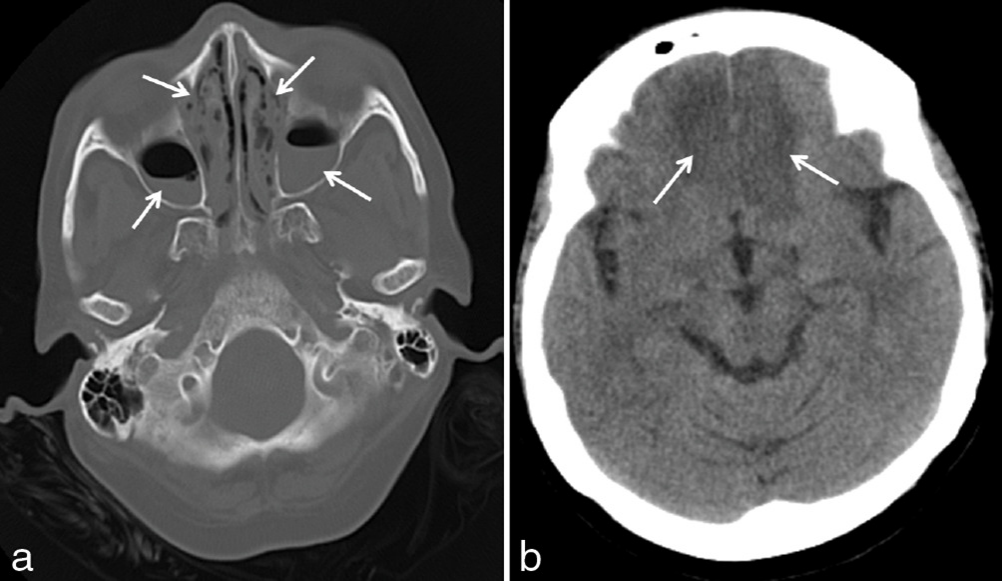
(a) Axial plain CT scan in bone window after 5 days showing worsening sinusitis with development of air–fluid levels in bilateral maxillary sinuses (arrows). (b) Plain CT scan in the axial section at the level of the brain showing interval appearance of hypodense areas in bilateral inferior frontal lobes (arrows).

**Figure 3.**
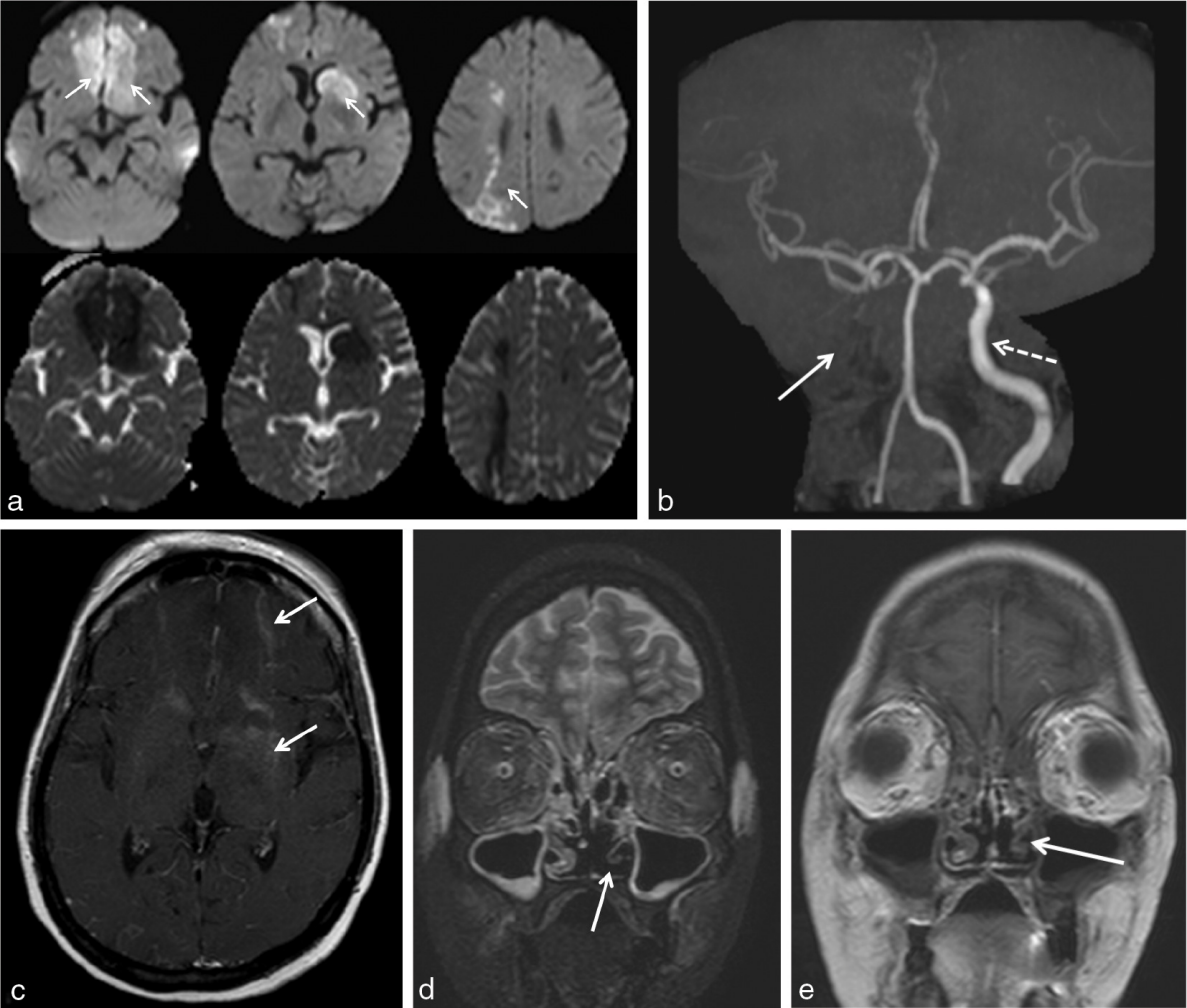
(a) Diffusion-weighted images with corresponding apparent diffusion coefficient maps shows multiple acute infarcts (arrows) in bilateral inferior frontal, left basal ganglia and right posterior watershed region. (b) MR angiography using non-contrast time-of-flight shows complete thrombosis of the right internal carotid artery (arrow). Broken arrow showing normal left internal carotid artery. (c) Axial contrast-enhanced *T*_1_ weighted image showing faint enhancement of the infarcts (arrows). (d) Short tau inversion-recovery in coronal showing absent normal mucosal hyperintensity (arrow) covering the left inferior turbinate (in discrepant with the mucosal thickening on the CT scan). (e) Coronal contrast-enhanced *T*_1_ (non-fat-saturated) weighted image shows non-enhancement of the left inferior turbinate mucosa (arrow)

2 days later, she was admitted to the emergency department with severe headache, altered sensorium and orbital swelling. Clinically, neurological deficits were documented. Laboratory tests at the time of admission were: random blood glucose 477 mg dl^–1^ (normal 70–140 mg dl^–1^); glycosylated haemoglobin 18.8%; C-reactive protein 228 mg l^–1^ (normal < 5 mg l^–1^); neutrophilic leukocytosis and thrombocytosis on peripheral smear; urine and cerebrospinal fluid were positive for protein, glucose and ketones. Plain CT scan followed by contrast-enhanced MRI using Gadodiamide (Omniscan 0.5 mmol ml^–1^, GE Healthcare) was performed, which showed worsening of the sinusitis (Figure 2a) with development of new hypodense areas in the bilateral frontal lobes (Figure 2b) and the left caudate nucleus, which was suggestive of infarcts. The contrast-enhanced MRI (Figure 3) revealed acute infarcts in the bilateral frontal lobes, right anterior and posterior watershed regions, left basal ganglia and left centrum semiovale (Figure 3a). The right intracranial ICA showed loss of normal signal void, with the MR angiography showing complete thrombosis of the right ICA (Figure 3b). Infarct in the left basal ganglia showed areas of blooming on gradient sequences. The infarcted areas also showed faint contrast enhancement (Figure 3c). Moderate mucosal thickening of all the paranasal sinuses was seen. Left medial orbital muscle and soft tissue oedema was noted corresponding to the CT images. The thickened mucosa lining the left middle and inferior turbinates appeared hypointense on the short tau inversion-recovery sequence and were non-enhancing on post-contrast administration (Figure 3d,e).

**Figure 4.**
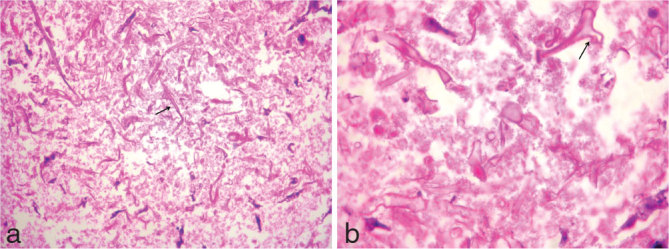
(a, b) Nasal biopsy with haematoxylin and eosin staining shows multiple broad, aseptate, irregular branching hyphae with obtuse angles, consistent with *Mucor* (arrows).

Based on the history of uncontrolled diabetes, worsening sinusitis with imaging features of multiple acute infarcts, which showed blooming and enhancement, thrombosed ophthalmic artery, occluded ICA, non-enhancing nasal turbinates, a diagnosis of angioinvasive rhino-orbito-cerebral mucormycosis was made. A nasal biopsy was performed, which showed branching hyphae with obtuse angles invading the vessels, consistent with mucormycosis (Figure 4a,b).

**Figure 5.**
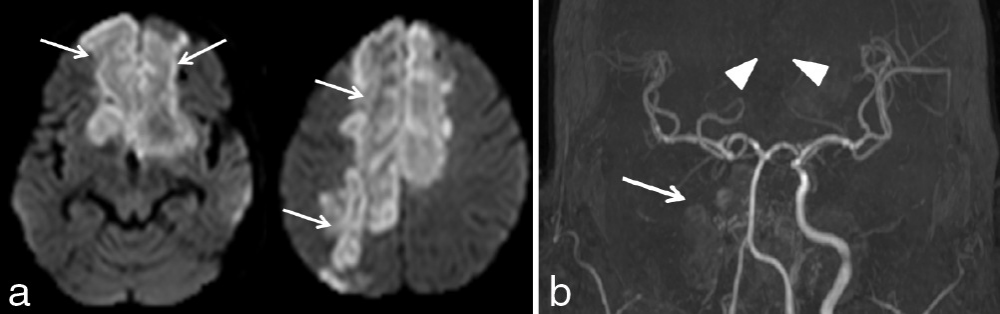
(a, b) MRI repeated after 2 days in view of deterioration despite treatment shows increasing infarcts (arrows in a) and additional thrombosis of bilateral anterior cerebral arteries (arrowheads in b).

The patient was vigorously treated with i.v. amphotericin and debridement, and control of hyperglycaemia, in spite of which she succumbed to death owing to massive evolving infarcts (Figure 5a, b).

## Discussion

Zygomycoses are fungal infections caused by organisms of the orders Entomophthorales and Mucorales.^1^ Mucorales are the causative agents of mucormycosis, a life-threatening infection afflicting the immunocompromised group. Among the six families of Mucorales, *Rhizopus, Mucor* and *Absidia* produce the infection frequently.^1^ Mucormycosis presents with rhino-orbito-cerebral, pulmonary, cutaneous, gastrointestinal and disseminated involvement among which the rhinocerebral form continues to be the most common manifestation.^2^ Approximately 70% of cases of rhinocerebral mucormycosis occurs in patients with uncontrolled diabetes mellites. Other underlying conditions include post-solid organ or stem cell transplantation and prolonged neutropenia owing to any cause.^3^ Occurrence in immunocompetant individuals is very rare and trauma with contaminated vegetative matter is many times the causative factor. The proposed reasons as to why there is increased risk of mycosis in diabetes include phagocytic dysfunction owing to hyperglycaemia and acidosis and due to increased serum iron levels in such patients, which help in the organism survival and pathogenicity.^1^ This group of fungi has more predilection for angioinvasiveness than other fungal groups such as aspergillosis and *Candida.* The spores of the *Mucor* invade the nasal and sinusal mucosa causing rhinosinusitis. Then it spreads to the orbit through the medial orbital wall or the nasolacrimal duct. It invades the walls of the arteries and veins, causing vascular mucor thrombosis, occlusion, infarction and subsequent spread to the intracranium, causing cerebral infarctions resulting in high mortality.^4,5^ Clinically, patients present with symptoms of sinusitis, fever, eye swelling, cranial nerve palsy and orbital apex syndrome. In cases of intracranial extension, patients can have altered sensorium and focal neurological deficits based on the region of spread.

Imaging features early in the course of the disease is non-specific, with CT showing rhinosinusal mucosal thickening, hyperdense contents and obliterated surrounding fat planes. Orbital spread can result in pre- and post-septal, retrobulbar and intraocular muscle soft tissue oedema. Frank bone destruction can be seen accompanied by bone sclerosis. But in angioinvasive forms, bone destruction need not be present as the spread is through blood vessels and foramina. In such cases, thrombosis of the involved vessels can be seen, as evident in this patient. On MRI, *T*_1_ and *T*_2_ weighted images show variable signal intensity.

Fungal elements appear hypointense on *T*_2_ images owing to the presence of iron and other minerals in them. The infarcted mucosa can show diffusion restriction owing to vascular thrombosis and does not show enhancement on gadolinium administration secondary to devitalization.^6,7^ This focal lack of enhancement of the involved mucosa owing to necrosis and devitalization in mucormycosis is referred to as the “black turbinate” sign.^8^ Complete thrombosis of the ICA is rare but has been reported^9^ in the literature. The *Mucor* thrombus extends retrogradely from the smaller ophthalmic arteries to involve the ICA in a short time span, complicating the already fulminating condition. Involvement of the ICA can additionally produce infarcts. The watershed infarcts in our case was secondary to ICA occlusion and other infarcts were secondary to direct vessel invasion from the rhino-orbito-sinusal disease. The infarcts due to direct angioinvasiveness actually harbour the fungal elements, which may explain blooming of such infarcts on gradient sequences representing the fungal elements itself.

Angioinvasive aspergillosis has similar presentation, imaging features and predisposing factors.^10^ Hence differentiation can be made only by histopathology, where the hyphae of *Aspergillus* show regular branching at acute angles and hyphae of *Mucor* show irregular branching at angles approaching 90° or more.

Other causes of infection-induced brain infarcts include vasculitis secondary to tuberculosis. In such cases, the infarcts are predominantly central (basal ganglia, thalami) owing to the propensity to involve the lenticulostriate and thalamostriate arteries. Additionally, ring-enhancing lesions representing a granuloma or abscess can be seen with or without enhancing meningeal exudates. Rhino-orbital disease is uncommon in such a scenario.

Another rare cause of rapidly progressing vascular thrombosis is the antiphospholipid antibody syndrome. The severe form of this entity is called “catastrophic” and causes multiorgan involvement secondary to thrombus formation developing over a short time.^11^ Treatment of rhinocerebral mucormycosis includes prompt control of hyperglycaemia, i.v. antifungals such as amphotericin and anti-oedema measures. Renal toxicity induced by amphotericin should be closely monitored with the use of alternative drugs in renal dysfunction. Surgical debridement of the necrotic tissue until normal perfused healthy tissue is seen can reduce the disease burden.

The uniqueness of mucormycosis is its spread to the intracranium through the arteries and veins without causing any bone or dural destruction. The common arteries of spread are the ophthalmic and ethmoidal arteries. The presence of ophthalmic artery thrombosis in the setting of sinusitis with a “black turbinate” and uncontrolled diabetes is an early clincher for the diagnosis of angioinvasive fungal sinusitis. Management at such an early stage would reduce substantial morbidity.

Angioinvasive rhinocerebral mucormycosis is an acute fulminant infection caused by fungi of the order Mucorales that targets uncontrolled diabetics and other immunosupressed individuals. Early imaging features of angioinvasiveness include the presence of thrombosed vessels, especially in the orbital regions and the “black turbinate” sign representing devitalized mucosa. Intracranial extension carries a grave prognosis, with death reported in 90% of cases.^5^

## Learning points

1. Rhinocerebral mucormycosis is an acute severe, rapidly progressing, life-threatening condition affecting uncontrolled diabetics and immunocompromised individuals.
2. “Black turbinate” sign on MRI is useful in the diagnosis of fungal sinusitis.
3. Thrombosis of the ophthalmic vessels can suggest and predict angioinvasiveness and subsequent development of cerebral infarcts.
4. Cerebral involvement carries a grave prognosis and hence aggressive treatment is essential.

## Consent

The informed consent to publish this case was obtained and is held on record.
